# Evoked and entrained pupillary activity while moving to preferred tempo and beyond

**DOI:** 10.1016/j.isci.2024.111530

**Published:** 2024-12-04

**Authors:** Connor Spiech, Mikael Hope, Valentin Bégel

**Affiliations:** 1 RITMO Centre for Interdisciplinary Studies in Rhythm, Time and Motion, University of Oslo, Oslo, Norway; 2 Department of Psychology, University of Oslo, Oslo, Norway; 3 Department of Psychology, Concordia University, Montreal, QC, Canada; 4 Montreal Centre for Brain, Music and Sound (BRAMS), Montreal, QC, Canada; 5 Centre for Research in Brain, Language and Music (CRBLM), Montreal, QC, Canada; 6 Department of Musicology, University of Oslo, Oslo, Norway; 7 Institut des Sciences du Sport Santé de Paris (I3SP), URP 3625, Université Paris Cité, Paris, France

**Keywords:** Health sciences, Sensory neuroscience, Cognitive neuroscience

## Abstract

People synchronize their movements more easily to rhythms with tempi closer to their preferred motor rates than with faster or slower ones. More efficient coupling at one’s preferred rate, compared to faster or slower rates, should be associated with lower cognitive demands and better attentional entrainment, as predicted by dynamical system theories of perception and action. We show that synchronizing one’s finger taps to metronomes at tempi outside of their preferred rate evokes larger pupil sizes, a proxy for noradrenergic attention, relative to passively listening. This demonstrates that synchronizing is more cognitively demanding than listening only at tempi outside of one’s preferred rate. Furthermore, pupillary phase coherence increased for all tempi while synchronizing compared to listening, indicating that synchronous movements resulted in more efficiently allocated attention. Beyond their theoretical implications, our findings suggest that rehabilitation for movement disorders should be tailored to patients’ preferred rates to reduce cognitive demands.

## Highlights

• Pupil activity was recorded when processing preferred, faster, and slower rates
• Only fast and slow rates evoke larger pupil sizes while synchronizing than listening
• Pupillary phase coherence increased while synchronizing compared to listening
• These results are not a simple product of movement

## Graphical abstract

**Figure:**
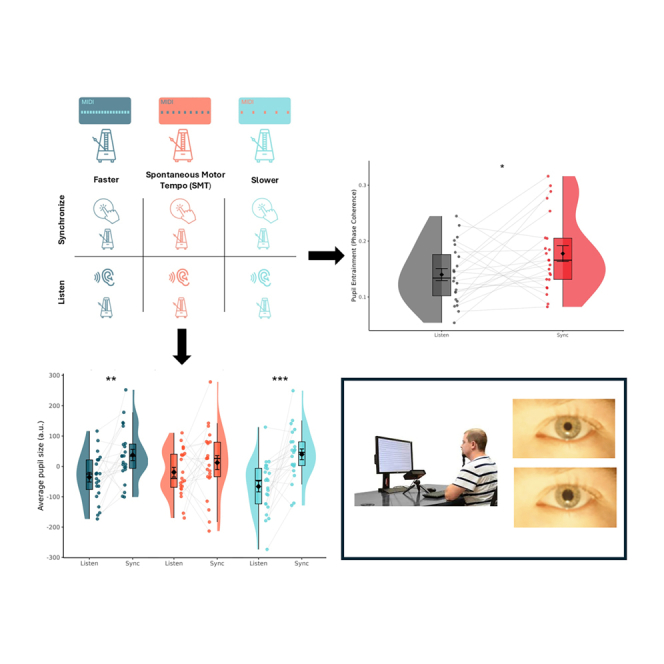

## Introduction

Rhythm is ubiquitous in human behavior: music and dance are inherently rhythmic, and most daily activities depend on finely timed motor control to coordinate our movement with the environment. Cognition also partly relies on rhythm, as evidenced by temporal predictability in language,^1^ and even memory and attention seem to be sustained by rhythmic brain processes.^2^^,^^3^^,^^4^ Reciprocally, rhythm processing is cognitively demanding; for example, stabilizing synchronization with increasingly complex rhythmic patterns requires additional attentional load.^5^^,^^6^^,^^7^^,^^8^^,^^9^

One prominent theory in psychology, dynamical systems theory, has sought to explain how the brain rhythmically deploys attention to the right moments in time. Dynamical systems theories assume that self-organized neural and behavioral systems coordinate perceptual and cognitive processes.^10^ Oscillator models are central mathematical and conceptual tools in dynamical system theories, and are particularly suited to study the anticipatory processes sustaining attentional and rhythmic mechanisms in human behavior.^11^^,^^12^^,^^13^ In these models, internal and behavioral oscillatory systems (e.g., electrical activity in populations of neurons, rhythmic movements like gait) synchronize to external events for more fluent processing.^14^^,^^15^^,^^16^^,^^17^^,^^18^^,^^19^

These oscillations appear spontaneously (i.e., in the absence of any external cues) at different rates for each individual.^20^ These spontaneous motor rhythms can be measured behaviorally by asking participants to tap at their own comfortable rate. Spontaneous rates have been shown to influence participants’ abilities to synchronize both to other people and music.^21^^,^^22^ Specifically, participants more optimally synchronize to rates that are closer to their own spontaneous rates. These findings may arise because smaller phase adjustments require fewer attentional resources or because oscillatory attention processes respond more efficiently via resonance-like properties. However, neither of these possible attentional mechanisms has yet been directly explored.

One simple yet reliable way to measure such attentional processes is via pupillary activity due to its tight coupling to noradrenergic activity from the locus coeruleus.^23^^,^^24^^,^^25^ Greater task-evoked pupil dilations and pupil size have consistently been linked to increased attention allocation since the 1960s^26^^,^^27^^,^^28^^,^^29^ and, more recently, oscillatory pupil activity (particularly in the delta range between ∼0.5 and 2 Hz) has been demonstrated to entrain to rhythms and appears to be related to movement.^30^^,^^31^^,^^32^ Unlike electroencephalography, the event-related pupil dilation response is so sluggish (typically peaking between ∼500 and 1,200 ms post-event onset^33^^,^^34^) that phase-aligned pupil dilations can be interpreted as entrained rather evoked. This is particularly relevant for testing theories like dynamical systems where the difference between evoked and entrained responses is crucial to the underlying neural mechanism.^35^^,^^36^^,^^37^^,^^38^^,^^39^ Thus, pupillometry offers a robust and convenient way to investigate (1) whether synchronizing to rates beyond one’s spontaneous rate demands more attention and (2) whether attention is better entrained (and therefore more efficiently processed according to dynamical systems theory) to one’s own spontaneous rate relative to faster and slower tempi.

**Figure 1:**
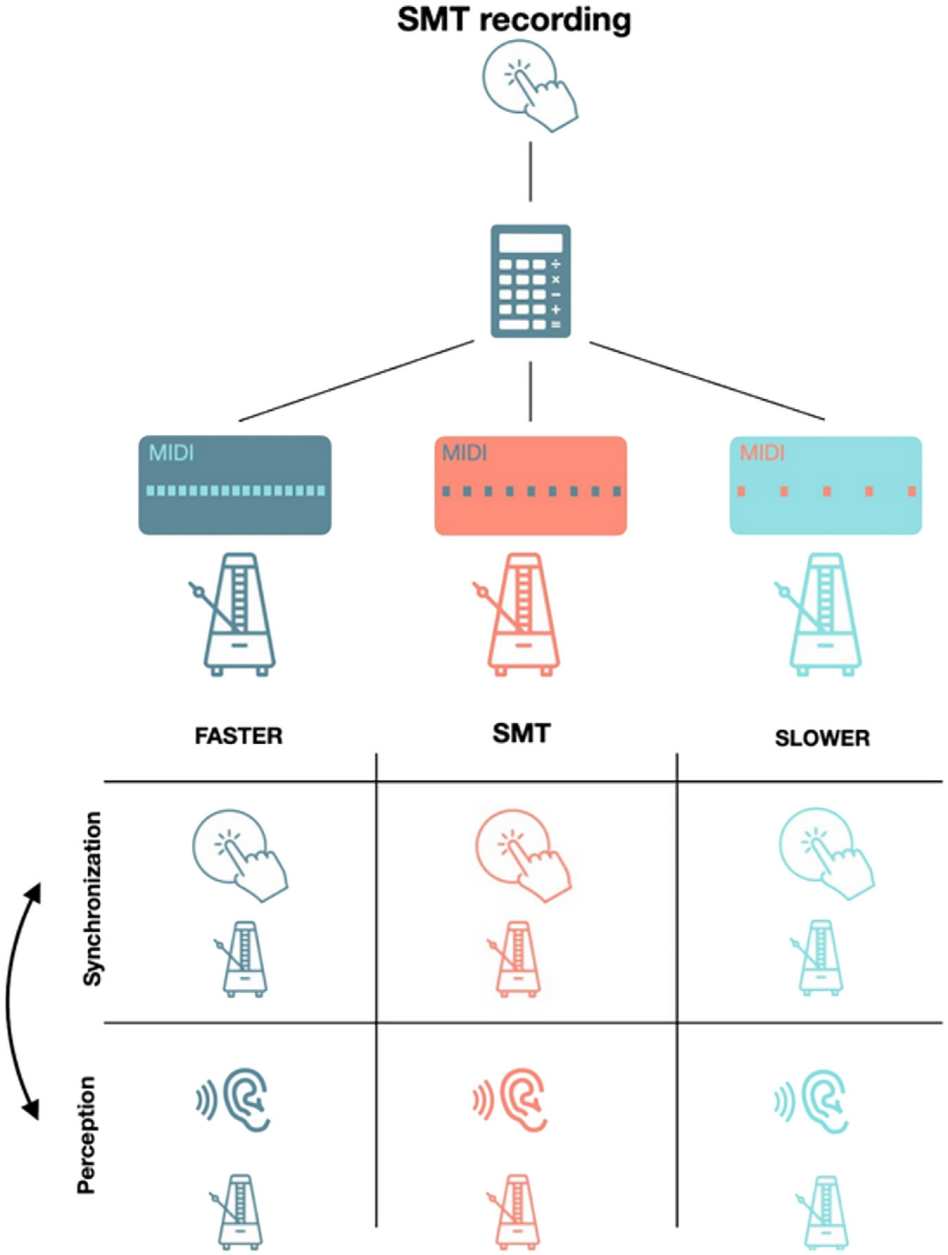
Experimental design The study design. Participants either tapped or listened to metronomes that matched their SMT or were 20% faster or slower. Half of the participants tapped to the metronomes first followed by listening to the metronomes (synchronization then perception) while the other half of the participants listened to the metronomes first followed by tapping to them (perception then synchronization).

To this end, we first recorded the spontaneous rates of 25 healthy adults in an unpaced tapping tapping (spontaneous motor tempo, SMT) and created three metronomes consisting of 60 woodblock sounds for each participant, one corresponding to their average SMT, one 20% faster, and one 20% slower than this rate. Participants were then tasked with either passively listening or synchronizing their finger taps to the metronomes across three trials per condition while their pupil size was recorded with an eye-tracker (Figure 1). We hypothesized that average pupil sizes would be larger while synchronizing relative to listening, particularly at the faster and slower tempi, reflecting increased attentional demands. Furthermore, we hypothesized that pupil activity would be better entrained (as assessed with phase coherence) at participants’ SMT where, according to dynamical systems theory, their internal oscillators are already primed to resonate for more efficient attentional processing.

## Results

**Figure 2:**
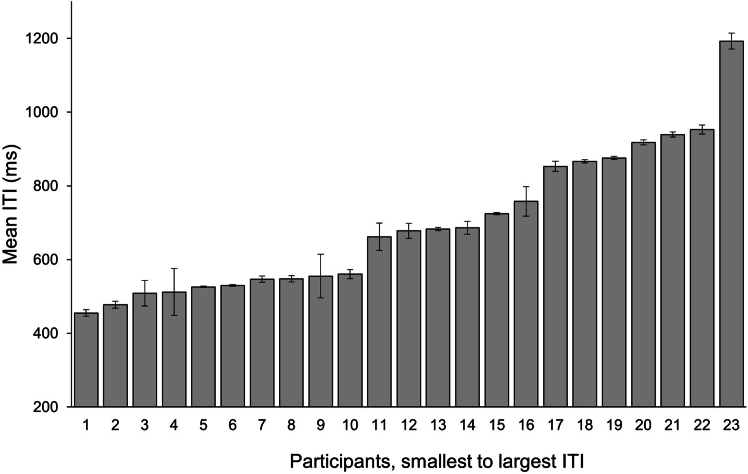
Distribution of participants’ SMTs Mean individual spontaneous motor tempi, ordered from fastest to slowest. Error bars indicate standard errors of the mean.

As in previous studies,^21^^,^^22^^,^^40^^,^^41^ participants’ SMTs spanned a relatively broad range from 462 to 1,192 ms (ITI, *M* = 692.34, *SD* = 190.34) (Figure 2). Despite this considerable variability, all participants nevertheless managed to synchronize their taps to each of their three custom metronomes with equal success as indicated by equivalent vector lengths (tapping consistency, *F*(1,44) = 1.37, *p* = 0.26, *η2G* = 0.11) and directions (tapping precision, *F*(1,44) = 2.42, *p* = 0.10, *η2G* = 0.13) in all tempo conditions. Thus, the following pupillometry results reflect the attentional processes employed to achieve this performance rather than attentional differences related to errors or their corrections.

### Synchronizing taps is more cognitively demanding than listening only beyond one’s SMT

**Figure 3:**
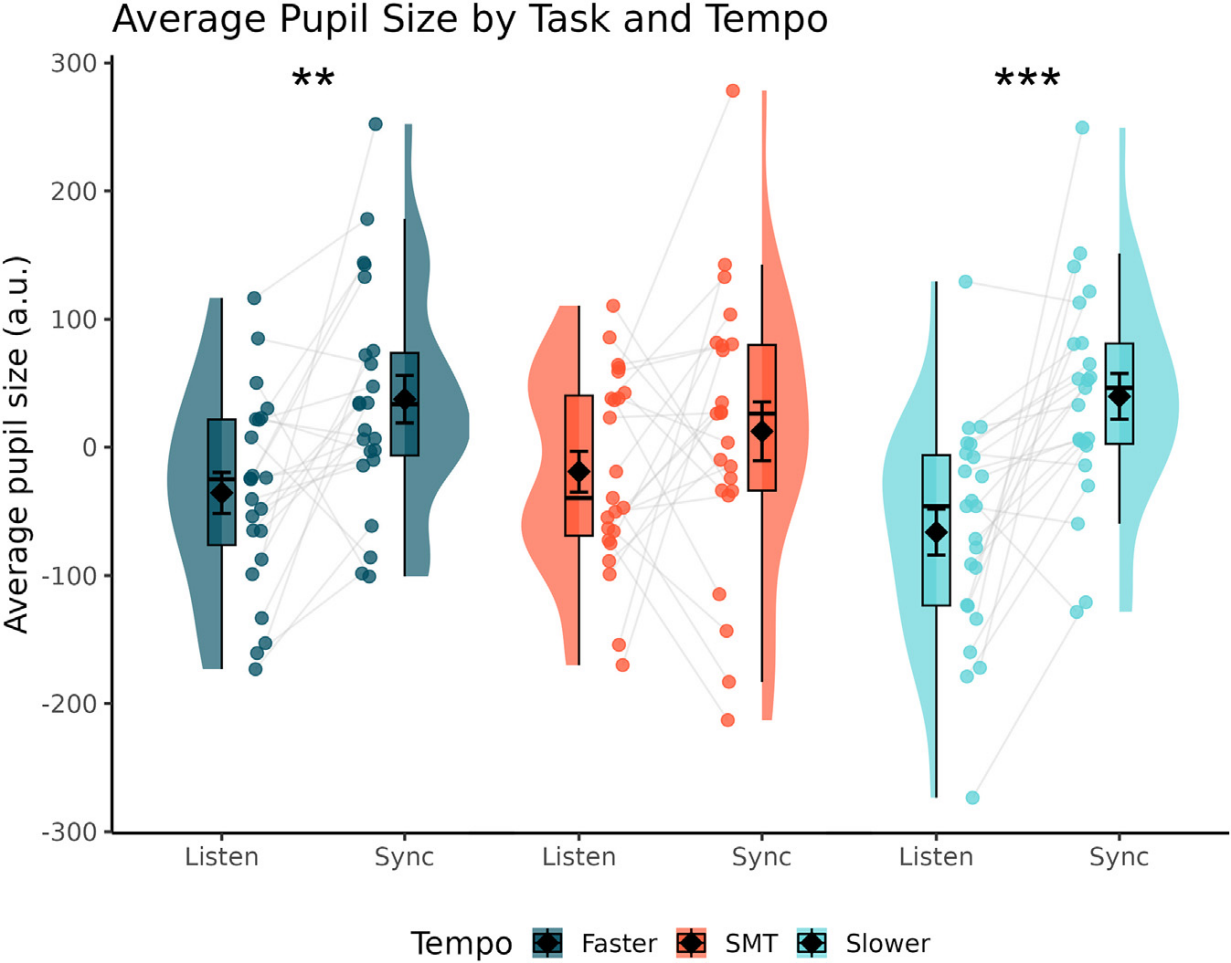
Evoked pupil size by task and tempo Raincloud plots for average pupil size by task and tempo condition. Black diamonds denote the mean while error bars represent one standard error of the mean. Each point depicts a single subject’s average pupil size and gray lines connect these averages between Task conditions. ∗∗*p* < 0.01, ∗∗∗*p* < 0.001 (post-hoc t tests).

Pupil size by task and tempo condition are presented in Figure 3. The repeated measures analysis of variance (ANOVA) on average task-evoked pupil sizes over the entire trial revealed a modest effect of task (*F*(1,22) = 12.530, *p* = 0.002, *η2G* = 0.142), indicating that synchronizing one’s taps to the metronome was more cognitively demanding than passively listening to the same metronome. Crucially, there was also an interaction with tempo (*F*(2,44) = 4.533, *p* = 0.016, *η2G* = 0.030) where post-hoc t tests demonstrated that the main effect of task was driven exclusively by the faster (*t*(22) = 3.301, *p* = 0.004, *d* = 0.69) and slower (*t*(22) = 4.434, *p* < 0.001, *d* = 0.92) metronomes evoking greater pupil sizes while synchronizing relative to listening whereas pupil sizes did not differ at their preferred rates (*t*(22) = 1.165, *p* = 0.257). These results show that synchronizing to the metronome only required more attention than listening to it when its tempo was outside of one’s comfortable rate.

### Synchronizing taps results in greater attentional entrainment at all tempi

**Figure 4:**
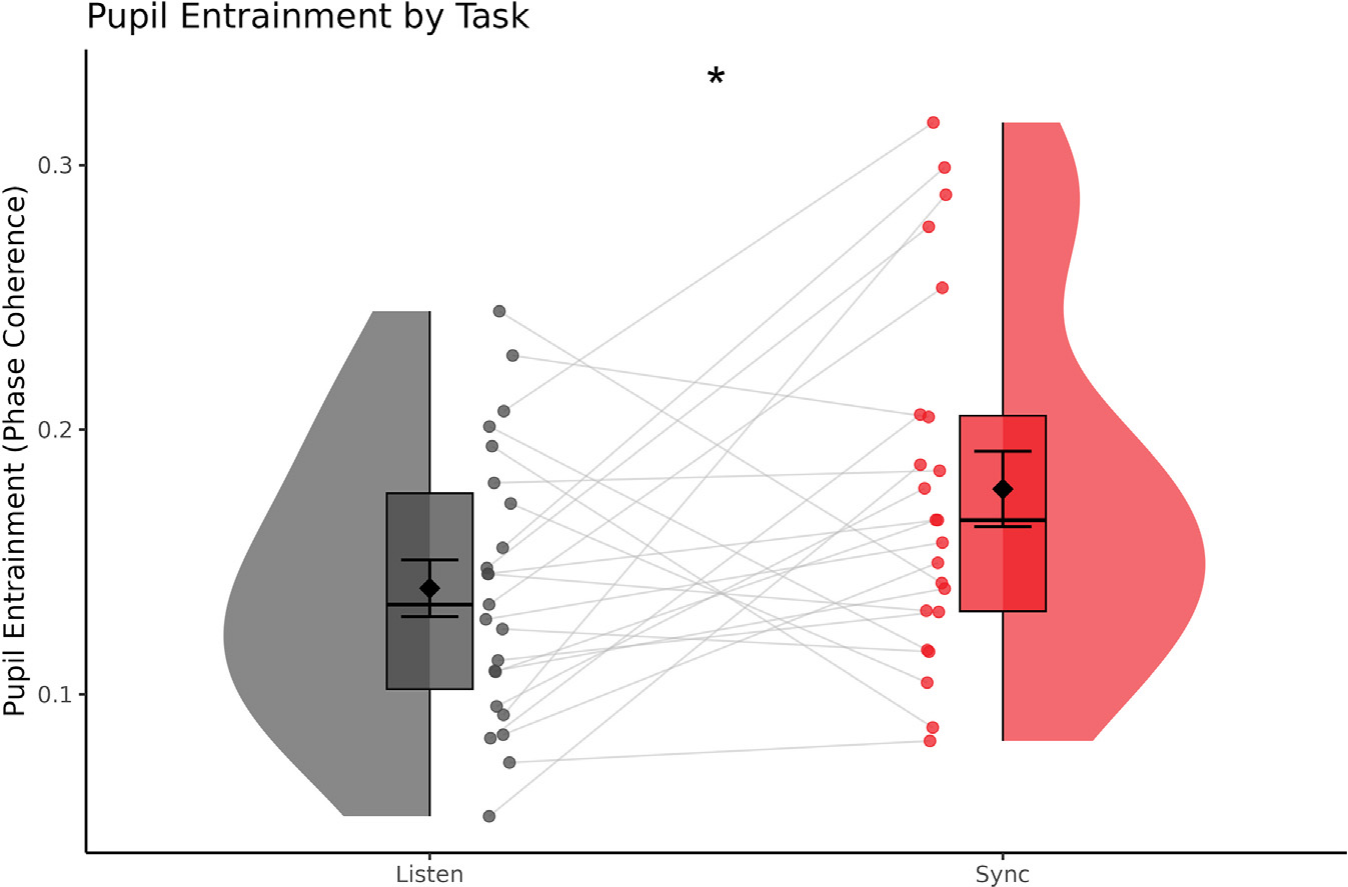
Pupillary entrainment by task Raincloud plots depicting the main effect of task on entrained pupil activity. Black diamonds denote the mean while error bars represent one standard error of the mean. Each point depicts a single subject’s average phase coherence and gray lines connect these averages between task conditions. ∗*p* < 0.05 (ANOVA).

An additional participant was missing too much data from the SMT condition, unbalancing the design, and thus had to be excluded. Nonetheless, the repeated measures ANOVA on entrained pupillary activity revealed a main effect of task (*F*(1,21) = 5.000, *p* = 0.036, *η2G* = 0.052) with no effect of tempo (*F*(2,42) = 1.131, *p* = 0.332) or interaction (*F*(2,42) = 0.932, *p* = 0.402). This demonstrates that attention was more efficiently deployed to the frequency of the metronome while participants were synchronizing their taps relative to passively listening (Figure 4).

### Evoked and entrained pupillary effects are not driven purely by movement

**Figure 5:**
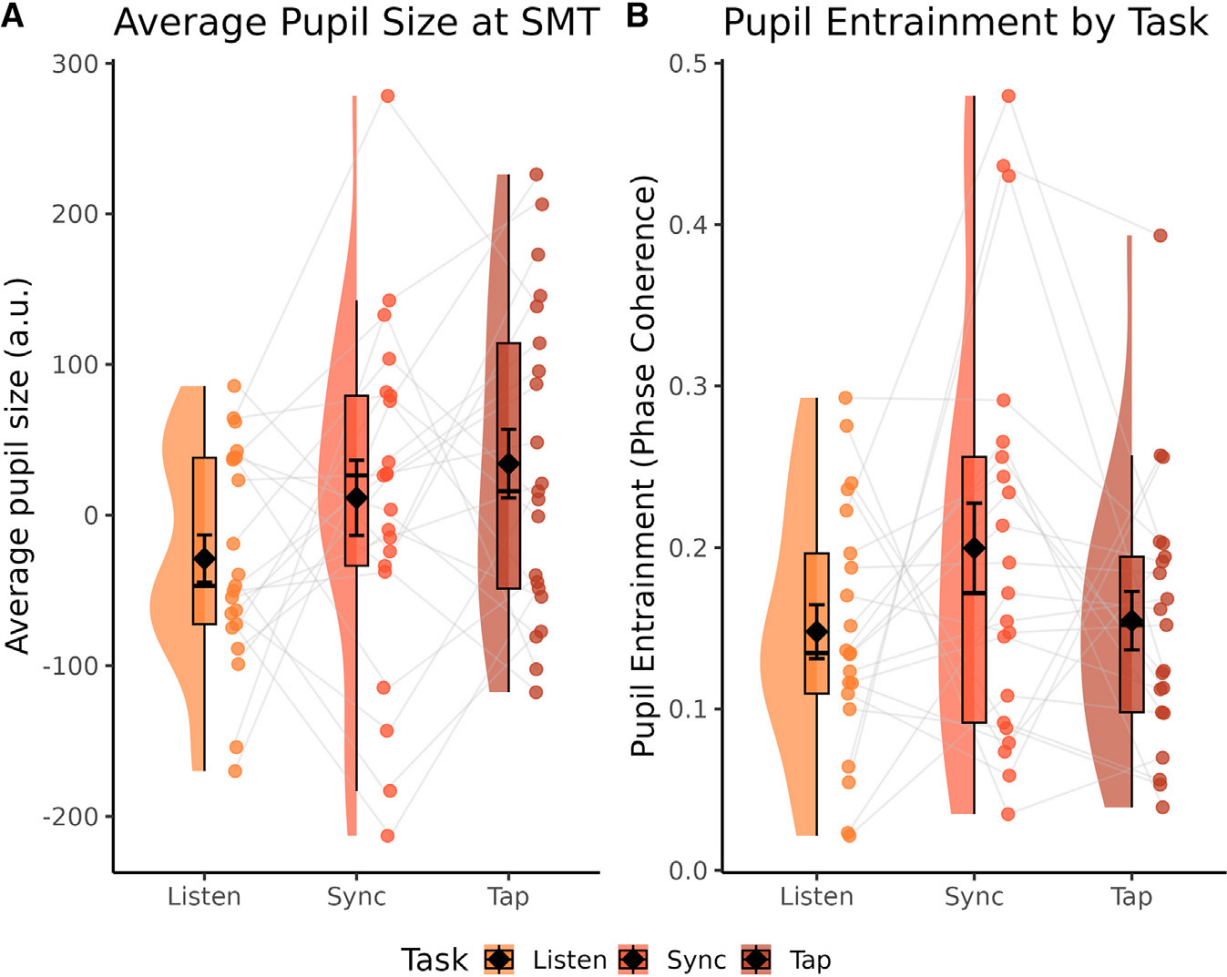
Control analyses Raincloud plots for the control analyses only on the SMT condition while listening, synchronizing, and tapping without a metronome. Black diamonds denote the mean while error bars represent one standard error of the mean. Each point depicts a single subject’s average pupil size (A) or phase coherence (B) and gray lines connect these averages between Task conditions. There were no significant differences observed.

One could argue that we might have observed the same pattern of results had we instructed participants to move without the presence of the metronome cues. That is to say that these effects could be mere motor effects rather than sensorimotor synchronization effects per se. As an additional control analysis, we analyzed participants’ average pupil dilations and entrained pupillary activity during their SMT recordings and compared this to their respective signals while listening or synchronizing to metronomes at this tempo. Two participants’ pupillary recordings during the SMT recordings were corrupted or missing and so were omitted from these control analyses. There was no effect of task on either the average evoked pupil size (*F*(2,40) = 2.514, *p* = 0.094) or pupillary phase coherence (*F*(2,40) = 1.823, *p* = 0.175). The results are plotted in Figure 5 in the following.

## Discussion

Here we tested whether synchronizing to metronomes at one’s preferred motor rate demands fewer cognitive resources (assessed by evoked pupil size) and whether it is associated with more efficient allocation of those resources (assessed by pupillary entrainment) compared to faster and slower rates. We found that synchronizing, relative to listening, evoked greater average pupil sizes at rates outside of participants’ preferred tempo, indicating that more attention was deployed to achieve the same level of synchronization to the faster and slower metronomes. Despite this overall increase in attention while synchronizing to faster and slower tempi, pupillary phase coherence was modestly greater while synchronizing compared to listening but in a similar manner across all tempi conditions.

Our average evoked pupil size results demonstrate that more attentional resources are allocated to synchronize to tempi outside of one’s comfortable rate, confirming our first hypothesis. This is consistent with previous results showing that more attentional resources are necessary for synchronizing with more complex stimuli, as reflected in behavioral and neurophysiological processes.^5^^,^^6^^,^^7^^,^^8^^,^^9^ Interestingly, these differences in attention allocation were not symmetrical; the effect was larger for slower metronomes (*d* = 0.90) than faster metronomes (*d* = 0.66). This asymmetry fits with previous studies where synchronization tends to be worse for slower tempi than faster tempi.^40^^,^^41^^,^^42^ This may be an effect of expertise, since the asymmetry seems to arise in nonmusicians more than in musicians who are more flexible in their ability to synchronize, including to slower tempi.^41^ Why this asymmetry exists, however, is still unclear. The precision of timing processing decreases with larger intervals (Weber’s law),^43^ leading to increased tapping variability and asynchronies at slower rates.^44^^,^^45^ The increased pupil size when tapping at slow rates may therefore reveal cognitive and attentional resources needed to overcome the natural tendency to tap with less precision and accuracy at slow tempi, as predicted by Weber’s law.

The oscillatory pupil activity results demonstrate that synchronous movements result in greater attentional entrainment. While most participants generally displayed a modest degree of entrainment even while listening, this entrainment was greater while synchronizing their taps to the metronomes. This is consistent with theories of active sensing and embodied cognition where action plays a central role in cognitive processes like attention.^46^^,^^47^^,^^48^^,^^49^ Contrary to our second hypothesis, there was no interaction with tempo, indicating that this motor effect is persistent across the range of tempi. Given participants’ successful behavioral synchronization at all tempi, this is rather unsurprising in hindsight, especially since all of our participants’ metronome rates fell well within the ecological range that bounds human rhythm perception and production.^50^^,^^51^ Thus, it seems that attentional deployment is more entrained for *successful* behavioral synchronization relative to passive listening.

Taken together, these results paint a more nuanced picture of dynamical systems theories of attention. As dynamical systems theories predict, synchronizing beyond one’s preferred tempo does seem to require more attention as evidenced by our evoked pupil dilation effects. However, our oscillatory pupillary activity results seem to contradict dynamical systems accounts based on resonance properties that would predict worse pupillary entrainment at tempi faster and slower than one’s SMT. It is possible that a reduction of pupillary entrainment would be observed at larger deviations from one’s SMT than the ones used in this study; this should be addressed in future experiments to test whether pupil oscillations are flexible or relatively rigid resonators that only entrain to a narrow set of frequencies. Entrained pupil dilations may also represent a form of active sensing, i.e., a top-down sampling routine that sharpens sensory representations of the environment.^47^^,^^52^^,^^53^^,^^54^ Under this view, the locus coeruleus may proactively release norepinephrine, dilating the pupils, to maximize the encoding of sensory information at attended moments in time, specifically those coinciding with our own movements, regardless of the motor rate.

Our study demonstrates the usefulness of considering participants’ comfortable rates in experimental settings where tempo is manipulated because there could be latent yet systematic differences in the attentional demands among subjects. This would be particularly important to consider in experiments where the complexity or difficulty of tracking the rhythm is of interest. For instance, there are well-known interactions between rhythmic complexity and tempo in both perception and synchronization^55^^,^^56^; these interactions may partially reflect the distance from participants’ SMT in addition to differences stemming from the physical stimulus rates. These considerations may be even more important outside of the lab, particularly in clinical settings for movement disorders. For instance, if existing rhythmic- and movement-based interventions for Parkinson’s disease are titrated to each individual’s preferred rate, these treatments could be less attentionally demanding to adhere to which could in turn boost retention. Our pupillary entrainment results may similarly have clinical implications for attention and developmental disorders. For example, both children and adults with attention deficit hyperactivity disorder (ADHD) display more variable tapping performance so pupillometry could provide a cheap, non-invasive biomarker for diagnosis alongside tapping behavior.^57^^,^^58^^,^^59^ Moreover, children with dyslexia have been found to have faster SMTs and more variable sensorimotor synchronization, indicating that pupillary biomarkers may exist for them as well.^60^^,^^61^^,^^62^ Additionally, because attention was better entrained while synchronizing, rhythmic interventions may offer a promising avenue for treating attention disorders.

### Conclusion

We provide evidence that the pupil signal can capture variations in attentional demands when synchronizing at different tempi. Consistent with modern theories of rhythmic attention such as dynamical systems and active sensing, we observed pupil oscillations aligned to the frequency of the perceived metronomes, with stronger alignment when movement coordination was required. These results thus demonstrate that pupillary activity is a useful measure of both attentional demands and attention allocation over time, providing opportunities for scientists and clinicians alike.

### Limitations of the study

One limitation of this work is that our analysis to control for purely motor (rather than synchronization) effects was limited to participants’ SMT tempo. It’s possible that moving without metronome cues at rates faster or slower than their comfortable rate could elicit results similar to our synchronization condition. However, were we to implement a movement only condition at the manipulated tempi, we would introduce a secondary task demand of moving at a faster or slower rate in addition to simply moving at a comfortable rate. This increased cognitive demand would induce larger pupil sizes, and thus confound a pure motor effect. Future work could investigate this possibility more directly using a synchronization-continuation paradigm where participants synchronize to a metronome cue and then continue tapping at that tempo after the metronome stops. That said, the continuation window would have to be long enough (e.g., longer than ∼4–10 s) for the pupil size to restabilize after the orienting response dilation from the metronome stopping, but not so long that the participants’ tapping rate drifts to a new tempo. Another limitation is that the influence of sex and gender was not tested in this study. The sample size was not sufficient to address this question. However, we do not expect that gender or sex would influence the results, as no studies on SMT and synchronization reported any effects from these factors.^63^

## Resource availability

### Lead contact

Further information and requests for resources and reagents should be directed to and will be fulfilled by the lead contact, Connor Spiech (connorrichard.spiech@concordia.ca).

### Materials availability

This study did not generate new materials.

### Data and code availability

• The data has been deposited at OSF: https://osf.io/7us96/?view_only=be3f4fd8cb1448129483295144b8f805 and is publicly available as of the date of publication. Accession numbers are listed in the key resources table.
• Code has been deposited at OSF: https://osf.io/7us96/?view_only=be3f4fd8cb1448129483295144b8f805 and is publicly available as of the date of publication. Accession numbers are listed in the key resources table.
• Any additional information required to reanalyze the data reported in this paper is available from the lead contact upon request.

## Acknowledgments

We thank the Joint Conference of the 17th International Conference on Music Perception and Cognition (ICMPC) and the 7th Conference of the Asia-Pacific Society for the Cognitive Sciences of Music (APSCOM) for publishing an article presenting preliminary results of this study and nominating the authors for a Young Researchers Award.

This work was supported by an Arnold Bentley New Initiatives Fund from the 10.13039/100014693Society for Education, Music and Psychology Research awarded to Valentin Bégel and Connor Spiech as well as by the 10.13039/501100005416Research Council of Norway through its Centres of Excellence scheme, project number 262762 and 10.13039/501100005366Universitetet i Oslo.

## Author contributions

Author V.B. conceived the study and all authors contributed to the design of the experiment. Authors M.H. and V.B. collected the data while authors V.B. and C.S. analyzed the tapping and pupillometry data, respectively. Visualizations of the data were generated by authors C.S. and M.H. with feedback from V.B. All authors contributed equally to the drafting and revision of the manuscript. Authors V.B. and C.S. acquired funding.

## Declaration of interests

The authors declare no competing interests.

## STAR★Methods

### Key resources table

**Table:** 

REAGENT or RESOURCE	SOURCE	IDENTIFIER
**Deposited data**		

Open Science Framework	Center for Open Science https://www.cos.io/	https://osf.io/7us96/?view_only=be3f4fd8cb1448129483295144b8f805

**Software and algorithms**		

MATLAB 2019a	Mathworks, Inc, MA, USA	https://www.mathworks.com/products/matlab.html
R 4.4.0	The R Project for Statistical Computing	https://www.r-project.org/
Reaper v. 6.45	Cockos Inc., NY, USA	https://www.reaper.fm/

### Experimental model and study participant details

Twenty-five healthy adults gave informed consent to participate in the study in accordance with the ethical approval granted by the Department of Psychology’s internal review board for research ethics at the University of Oslo (reference number 8131575). Participants were compensated with a 150 NOK (∼€15) gift card for their time. Two participants were discarded because their average SMT or average pupil size were outside the normal range of the sample (i.e., differing from the median of the sample by more than three standard deviations). The final sample of participants was thus composed of 23 participants (10 men, 13 women) with an average age of 28.22 years (range: 20–36, SD = 4.03 years).

### Method details

#### Equipment and stimuli

Tapping data were collected using a Roland V-drums snare. The auditory stimuli consisted of metronome sequences composed of 60 cues (using a woodblock sample) created with MATLAB 2019a. They were played at a comfortable volume from two Genelec speakers (model 8030W) using custom scripts in MATLAB. The auditory signal and the Midi signal were recorded by a Roland V-drums Sound Module TD17 connected to a Windows computer using Reaper v. 6.45 (Cockos Inc., NY, USA). Pupil size was continuously recorded at 500 Hz with an EyeLink Portable Duo and a chin rest 70 cm from the eye-tracker in a well-lit room.

#### Procedure

The experiment started with participants’ spontaneous motor tempi being recorded. To familiarize them with the procedure, the participant completed a short practice trial where they produced a sequence of ten taps at an uncued rate. Once they understood the task, they completed three experimental trials consisting of 60 taps. Each participant’s SMT was then calculated as the mean intertap interval (ITI, in ms) across the three SMT trials after the first ten taps were discarded from each trial. This value was used to create the metronomes; one metronome matched their SMT (SMT condition), one was 20% faster (Faster condition), and one was 20% slower (Slower condition). At the start of the main experiment, participants completed a five-point (cross-shaped) calibration procedure for the eye-tracker. Subsequently, one of the three metronome sequences were played, selected pseudo-randomly such that all three tempi were played before being randomly shuffled again. Participants were instructed to fixate within a black ring presented in the center of a gray background while either passively listening to the metronome or synchronizing their taps to the metronome with their dominant index finger. Participants completed nine trials of either listening or tapping before completing nine more trials of the other for a total of 18 trials with the task order counterbalanced across subjects. Thus, the experiment followed a within-subject design with Tempo (SMT, Faster, Slower) and Task (Listen, Tap) as factors with three trials per condition. The study design is schematized in Figure 5 below.

### Quantification and statistical analysis

Each finger tap was matched with the nearest metronome cue. Signed asynchrony values were calculated as the participant’s tap onset time minus the auditory cued onset time (in ms). A negative asynchrony value indicates that the tap occurred before the cue while a positive value indicates that the tap occurred after the cue time. The first four taps of each sequence were also discarded to allow for tempo stabilization as is standard procedure. Some taps (i.e., when participants tapped too softly) were not captured by the midi touchpad. Therefore, a minimum of forty taps was necessary to analyze a trial; ten trials (out of 225, 4.4%) were discarded because there were fewer than 40 recorded taps. Finally, outliers, defined as taps with signed asynchronies of more than three standard deviations from the mean signed asynchrony in a trial, were discarded (fewer than 0.5% of the taps in total). There was at least one trial per condition for each participant after these data cleaning procedure.

In order to compare the magnitude and variability of the asynchronies across tempi conditions, circular statistics^64^^,^^65^ were conducted on the synchronization data using the MATLAB Circular Statistics Toolbox.^66^ The interval between metronome cues was represented on a 360° circular scale, and individual taps were represented as unit vectors with a given angle. A mean resultant vector *R* was calculated based on each individual tap in a trial. The length of the resultant vector, between zero and one, indicates synchronization consistency (i.e., the reciprocal of variability; the closer to one, the less variable the performance). The angle of the vector (in degrees) represents the relative phase, an index of synchronization accuracy. Negative angles indicate that participants tapped before metronome cue.

Pupil data were exported offline from Eyelink DataViewer^67^ for preprocessing with custom R scripts using functions from the “pupillometry” and “gazeR” packages in R.^68^^,^^69^ First, the timeseries for each participant’s right pupil was read in and locked to the stimuli onsets. Blinks were removed along with their preceding and succeeding 100 ms to exclude artifacts arising from partial occlusions of the pupil by the eyelid during a blink. Each trial was then smoothed using a 4 ms moving average and gaps smaller than 1000 ms were interpolated using cubic splines. Afterward, each trial was baseline-corrected by subtracting the median pupil value recorded during the 1000 ms of silence preceding stimuli onsets to remove random fluctuations in pupil size between trials.^24^ At this point, surviving artifacts were removed in a tripartite fashion: trials with more than 33% of missing data were discarded, samples containing rapid pupil size changes were detected and eliminated using the median absolute deviation of the dilation speed time series and a constant of 16 as suggested by Kret & Sjak-Shie,^70^ and visual inspection of a histogram of all remaining pupil sizes as recommended by Mathôt and colleagues.^71^ Each subject’s cleaned pupil size was averaged over the entire trial.

To investigate pupillary entrainment, additional processing was needed. First, the data from the first four taps needed to be discarded because of the orienting responses elicited by the metronome onset. Next, each trial for each subject was high-pass filtered with a third order Butterworth filter at 0.05 Hz to remove the signal drift, and then the data were z-scored according to the preprocessing steps employed and validated in past research.^30^^,^^31^ The time series for the remaining 56 taps were grouped into 14 four-tap phrases and timelocked to the start of each phrase for greater statistical power. Fourier series at the frequency of the metronome in each condition were extracted from each phrase in each trial using a fast Fourier transform.^72^ Three neighboring frequency bins were kept as well to control for spectral leakage as done in past work.^73^ As in previous studies,^74^^,^^75^ pupillary phase coherence was calculated by dividing the Fourier coefficients by their absolute values to get the phase angles, summing the resulting phase angles, and then dividing them by the total number of phrases per trial, and then taking the absolute value of the complex mean. The complex mean was then normalized, so that it can be compared across tempi (between zero and one, the closer to one, the less variable the performance).

Synchronization (Vector Length, Vector Direction) and pupillometry (Pupil Size, Phase Coherence) indices were used as the dependent variables in a three (Faster, SMT, Slower) by two (Listen, Tap) repeated measures ANOVA. Following a significant interaction, Welch’s paired sample t-tests were conducted and corrected for multiple comparisons using the false discovery rate method.^76^ In all figures, significance is denoted with a single asterisk for *p* < 0.05, a double asterisk for *p* < 0.01, and a triple asterisk for *p* < 0.001.

## References

[1] Kotz S.A., Schmidt-Kassow M. (2015). Basal ganglia contribution to rule expectancy and temporal predictability in speech. Cortex.

[2] Fiebelkorn I.C., Kastner S. (2019). A Rhythmic Theory of Attention. Trends Cogn. Sci..

[3] Köster M., Gruber T. (2022). Rhythms of human attention and memory: An embedded process perspective. Front. Hum. Neurosci..

[4] Lakatos P., Karmos G., Mehta A.D., Ulbert I., Schroeder C.E. (2008). Entrainment of neuronal oscillations as a mechanism of attentional selection. Science.

[5] Temprado J.-J., Laurent M. (2004). Attentional load associated with performing and stabilizing a between-persons coordination of rhythmic limb movements. Acta. Psychol..

[6] Temprado J.-J., Zanone P.-G., Monno A., Laurent M. (1999). Attentional load associated with performing and stabilizing preferred bimanual patterns. J. Exp. Psychol. Hum. Percept. Perform..

[7] Coull J.T., Nobre A.C. (1998). Where and when to pay attention: the neural systems for directing attention to spatial locations and to time intervals as revealed by both PET and fMRI. J. Neurosci..

[8] Coull J.T., Frith C.D., Büchel C., Nobre A.C. (2000). Orienting attention in time: behavioural and neuroanatomical distinction between exogenous and endogenous shifts. Neuropsychologia.

[9] Versaci L., Laje R. (2021). Time-oriented attention improves accuracy in a paced finger-tapping task. Eur. J. Neurosci..

[10] Kelso J. (1995).

[11] Haken H., Kelso J.A., Bunz H. (1985). A theoretical model of phase transitions in human hand movements. Biol. Cybern..

[12] Roman I.R., Washburn A., Large E.W., Chafe C., Fujioka T. (2019). Delayed feedback embedded in perception-action coordination cycles results in anticipation behavior during synchronized rhythmic action: A dynamical systems approach. PLoS Comput. Biol..

[13] Stepp N., Turvey M.T. (2010). On strong anticipation. Cogn. Syst. Res..

[14] Jones M.R. (1976). Time, our lost dimension: Toward a new theory of perception, attention, and memory. Psychol. Rev..

[15] Jones M.R. (2018).

[16] Jones M.R., Boltz M. (1989). Dynamic attending and responses to time. Psychol. Rev..

[17] Large E.W., Herrera J.A., Velasco M.J. (2015). Neural Networks for Beat Perception in Musical Rhythm. Front. Syst. Neurosci..

[18] Large E.W., Jones M.R. (1999). The dynamics of attending: How people track time-varying events. Psychol. Rev..

[19] Large E.W., Snyder J.S. (2009). Pulse and meter as neural resonance. Ann. N. Y. Acad. Sci..

[20] Desbernats A., Martin E., Tallet J. (2023). Which factors modulate spontaneous motor tempo? A systematic review of the literature. Front. Psychol..

[21] Zamm A., Wellman C., Palmer C. (2016). Endogenous rhythms influence interpersonal synchrony. J. Exp. Psychol. Hum. Percept. Perform..

[22] Zamm A., Wang Y., Palmer C. (2018). Musicians’ natural frequencies of performance display optimal temporal stability. J. Biol. Rhythms..

[23] Endestad T., Godøy R.I., Sneve M.H., Hagen T., Bochynska A., Laeng B. (2020). Mental Effort When Playing, Listening, and Imagining Music in One Pianist’s Eyes and Brain. Front. Hum. Neurosci..

[24] Laeng B., Alnaes D. (2019). Eye Movement Research.

[25] Murphy P.R., O’Connell R.G., O’Sullivan M., Robertson I.H., Balsters J.H. (2014). Pupil diameter covaries with BOLD activity in human locus coeruleus. Hum. Brain Mapp..

[26] Alnæs D., Sneve M.H., Espeseth T., Endestad T., van de Pavert S.H.P., Laeng B. (2014). Pupil size signals mental effort deployed during multiple object tracking and predicts brain activity in the dorsal attention network and the locus coeruleus. J. Vis..

[27] Kahneman D., Beatty J. (1966). Pupil diameter and load on memory. Science.

[28] Beatty J., Kahneman D. (1966). Pupillary changes in two memory tasks. Psychon. Sci..

[29] Oliva M. (2019). Pupil size and search performance in low and high perceptual load. Cogn. Affect. Behav. Neurosci..

[30] Fink L.K., Hurley B.K., Geng J.J., Janata P. (2018). A linear oscillator model predicts dynamic temporal attention and pupillary entrainment to rhythmic patterns. J. Eye Mov. Res..

[31] Spiech C., Danielsen A., Laeng B., Endestad T. (2024). Oscillatory attention in groove. Cortex.

[32] Vidal M., Onderdijk K.E., Aguilera A.M., Six J., Maes P.J., Fritz T.H., Leman M. (2024). Cholinergic-related pupil activity reflects level of emotionality during motor performance. Eur. J. Neurosci..

[33] Hoeks B., Levelt W.J.M. (1993). Pupillary dilation as a measure of attention: A quantitative system analysis. Behav. Res. Methods Instrum. Comput..

[34] McCloy D.R., Larson E.D., Lau B., Lee A.K.C. (2016). Temporal alignment of pupillary response with stimulus events via deconvolution. J. Acoust. Soc. Am..

[35] Lenc T., Keller P.E., Varlet M., Nozaradan S. (2018). Neural tracking of the musical beat is enhanced by low-frequency sounds. Proc. Natl. Acad. Sci. USA.

[36] Lenc T., Keller P.E., Varlet M., Nozaradan S. (2018). Reply to Novembre and Iannetti: Conceptual and methodological issues. Proc. Natl. Acad. Sci. USA.

[37] Lenc T., Keller P.E., Varlet M., Nozaradan S. (2019). Reply to Rajendran and Schnupp: Frequency tagging is sensitive to the temporal structure of signals. Proc. Natl. Acad. Sci. USA.

[38] Novembre G., Iannetti G.D. (2018). Tagging the musical beat: Neural entrainment or event-related potentials?. Proc. Natl. Acad. Sci. USA.

[39] Rajendran V.G., Schnupp J.W.H. (2019). Frequency tagging cannot measure neural tracking of beat or meter. Proc. Natl. Acad. Sci. USA.

[40] Bégel V., Demos A.P., Wang M., Palmer C. (2022). Social Interaction and Rate Effects in Models of Musical Synchronization. Front. Psychol..

[41] Scheurich R., Zamm A., Palmer C. (2018). Tapping Into Rate Flexibility: Musical Training Facilitates Synchronization Around Spontaneous Production Rates. Front. Psychol..

[42] Bégel V., Demos A.P., Palmer C. (2024). Duet synchronization interventions affect social interactions. Sci. Rep..

[43] Killeen P.R., Weiss N.A. (1987). Optimal timing and the Weber function. Psychol. Rev..

[44] Repp B.H., Su Y.H. (2013). Sensorimotor synchronization: a review of recent research (2006–2012. Psychon. Bull. Rev..

[45] Repp B.H., Doggett R. (2007). Tapping to a Very Slow Beat: A Comparison of Musicians and Nonmusicians. Music Percept..

[46] Clark A. (1999). An embodied cognitive science?. Trends. Cogn. Sci..

[47] Morillon B., Schroeder C.E., Wyart V. (2014). Motor contributions to the temporal precision of auditory attention. Nat. Commun..

[48] Shapiro L. (2019).

[49] Zalta A., Petkoski S., Morillon B. (2020). Natural rhythms of periodic temporal attention. Nat. Commun..

[50] London J. (2012).

[51] Repp B.H. (2005). Sensorimotor synchronization: A review of the tapping literature. Psychon. Bull. Rev..

[52] Calderone D.J., Lakatos P., Butler P.D., Castellanos F.X. (2014). Entrainment of neural oscillations as a modifiable substrate of attention. Trends Cogn. Sci..

[53] Lakatos P., Gross J., Thut G. (2019). A New Unifying Account of the Roles of Neuronal Entrainment. Curr. Biol..

[54] Schroeder C.E., Wilson D.A., Radman T., Scharfman H., Lakatos P. (2010). Dynamics of active sensing and perceptual selection. Curr. Opin. Neurobiol..

[55] Etani T., Marui A., Kawase S., Keller P.E. (2018). Optimal Tempo for Groove: Its Relation to Directions of Body Movement and Japanese nori. Front. Psychol..

[56] Matthews T.E., Thibodeau J.N.L., Gunther B.P., Penhune V.B. (2016). The Impact of Instrument-Specific Musical Training on Rhythm Perception and Production. Front. Psychol..

[57] Kliger Amrani A., Zion Golumbic E. (2020). Spontaneous and stimulus-driven rhythmic behaviors in ADHD adults and controls. Neuropsychologia.

[58] Gustafsson P., Kjell K., Cundari M., Larsson M., Edbladh J., Madison G., Kazakova O., Rasmussen A. (2023). The ability to maintain rhythm is predictive of ADHD diagnosis and profile. BMC. Psychiatry..

[59] Rubia K., Taylor A., Taylor E., Sergeant J.A. (1999). Synchronization, anticipation, and consistency in motor timing of children with dimensionally defined attention deficit hyperactivity behaviour. Percept. Mot. Skills..

[60] Bégel V., Dalla Bella S., Devignes Q., Vandenbergue M., Lemaître M.-P., Dellacherie D. (2022). Rhythm as an independent determinant of developmental dyslexia. Dev. Psychol..

[61] Goswami U. (2011). A temporal sampling framework for developmental dyslexia. Trends Cogn. Sci..

[62] Puyjarinet F., Bégel V., Lopez R., Dellacherie D., Dalla Bella S. (2017). Children and adults with Attention-Deficit/Hyperactivity Disorder cannot move to the beat. Sci. Rep..

[63] Desbernats A., Martin E., Tallet J. (2023). Which factors modulate spontaneous motor tempo? A systematic review of the literature. Front. Psychol..

[64] Dalla Bella S., Farrugia N., Benoit C.-E., Begel V., Verga L., Harding E., Kotz S.A. (2017). BAASTA: Battery for the assessment of auditory sensorimotor and timing abilities. Behav. Res. Methods.

[65] Fisher N.I. (1995).

[66] Berens P. (2009). CircStat: a MATLAB toolbox for circular statistics. J. Stat. Softw..

[67] EyeLink Data Viewer (2018). Version 3.2.1 (SR Research Ltd.). https://www.sr-research.com/data-viewer/.

[68] Geller J., Winn M.B., Mahr T., Mirman D. (2020). GazeR: A Package for Processing Gaze Position and Pupil Size Data. Behav. Res. Methods.

[69] Tsukahara J.S. (2022). pupillometry: An R package to preprocess pupil data (v0.8.1).

[70] Kret M.E., Sjak-Shie E.E. (2019). Preprocessing pupil size data: Guidelines and code. Behav. Res. Methods.

[71] Mathôt S., Fabius J., Van Heusden E., Van der Stigchel S. (2018). Safe and sensible preprocessing and baseline correction of pupil-size data. Behav. Res. Methods.

[72] Cooley J.W., Tukey J.W. (1965). An algorithm for the machine calculation of complex Fourier series. Math. Comput..

[73] Nozaradan S., Peretz I., Missal M., Mouraux A. (2011). Tagging the neuronal entrainment to beat and meter. J. Neurosci..

[74] Tallon-Baudry C., Bertrand O., Delpuech C., Pernier J. (1996). Stimulus specificity of phase-locked and non-phase-locked 40 Hz visual responses in human. J. Neurosci..

[75] Van Diepen R.M., Mazaheri A. (2018). The Caveats of observing Inter-Trial Phase-Coherence in Cognitive Neuroscience. Sci. Rep..

[76] Benjamini Y., Hochberg Y. (1995). Controlling the False Discovery Rate: A Practical and Powerful Approach to Multiple Testing. J. R. Stat. Soc. Ser. B Methodol..

